# Influenza A viruses in gulls in landfills and freshwater habitats in Minnesota, United States

**DOI:** 10.3389/fgene.2023.1172048

**Published:** 2023-05-09

**Authors:** Elizabeth A. Rasmussen, Agata Czaja, Francesca J. Cuthbert, Gene S. Tan, Philippe Lemey, Martha I. Nelson, Marie R. Culhane

**Affiliations:** ^1^ Department of Veterinary Population Medicine, College of Veterinary Medicine, University of Minnesota, Saint Paul, MN, United States; ^2^ Computational Biology Branch, National Center for Biotechnology Information, National Library of Medicine, National Institutes of Health, Bethesda, MD, United States; ^3^ Department of Fisheries, Wildlife and Conservation Biology, College of Food, Agricultural and Natural Resource Sciences, University of Minnesota, Minneapolis, MN, United States; ^4^ J. Craig Venter Institute, La Jolla, Division of Infectious Diseases, Department of Medicine, University of California San Diego, La Jolla, CA, United States; ^5^ Department of Microbiology, Immunology and Transplantation, Rega Institute, KU Leuven, Leuven, Belgium

**Keywords:** minnesota, migration, freshwater, ring-billed gull, franklin's gull, influenza

## Abstract

**Introduction:** The unpredictable evolution of avian influenza viruses (AIVs) presents an ongoing threat to agricultural production and public and wildlife health. Severe outbreaks of highly pathogenic H5N1 viruses in US poultry and wild birds since 2022 highlight the urgent need to understand the changing ecology of AIV. Surveillance of gulls in marine coastal environments has intensified in recent years to learn how their long-range pelagic movements potentially facilitate inter-hemispheric AIV movements. In contrast, little is known about inland gulls and their role in AIV spillover, maintenance, and long-range dissemination.

**Methods:** To address this gap, we conducted active AIV surveillance in ring-billed gulls (*Larus delawarensis*) and Franklin's gulls (*Leucophaeus pipixcan*) in Minnesota's natural freshwater lakes during the summer breeding season and in landfills during fall migration (1,686 samples).

**Results:** Whole-genome AIV sequences obtained from 40 individuals revealed three-lineage reassortants with a mix of genome segments from the avian Americas lineage, avian Eurasian lineage, and a global “Gull” lineage that diverged more than 50 years ago from the rest of the AIV global gene pool. No poultry viruses contained gull-adapted H13, NP, or NS genes, pointing to limited spillover. Geolocators traced gull migration routes across multiple North American flyways, explaining how inland gulls imported diverse AIV lineages from distant locations. Migration patterns were highly varied and deviated far from assumed “textbook” routes.

**Discussion:** Viruses circulating in Minnesota gulls during the summer breeding season in freshwater environments reappeared in autumn landfills, evidence of AIV persistence in gulls between seasons and transmission between habitats. Going forward, wider adoption of technological advances in animal tracking devices and genetic sequencing is needed to expand AIV surveillance in understudied hosts and habitats.

## Introduction

Aquatic birds are highly diverse in morphology, ecology, and behavior, inhabiting all seven continents and encompassing at least ten taxological orders, including Anseriformes (e.g., ducks and geese) and Charadriiformes (e.g., shorebirds and gulls) that are well-established reservoir hosts for avian influenza viruses (AIVs) (Slemons et al., 1974). Since the 1970s, tens of thousands of AIV specimens have been collected globally (Shu and McCauley, 2017), but large gaps remain in our understanding of the virus’s ecology and long-range movements. Historically, AIV surveillance has targeted waterfowl (e.g., ducks and geese (Jourdain et al., 2010), shorebirds (e.g., ruddy turnstones (Poulson et al., 2017), and poultry (Pittman et al., 2007). In recent years, more attention has been paid to gulls and how their long-range movements span multiple avian flyways and spread AIVs over long distances, specifically species in marine coastal environments (Anderson et al., 2020; Hill et al., 2022; Ineson et al., 2022). North America has four north-south flyways (Pacific, Central, Mississippi, Atlantic) separated by natural landmarks (mountain ranges, lakes, etc.) (Lincoln, 1935; Bellrose, 1968). The flyways capture the general migration movements of birds between southern wintering areas and northern breeding grounds and are a useful tool for ecological research and wildlife management. However, the concept oversimplifies the varied (and often east-west) movements of many species, especially those in abundance such as gulls, and epizootics—including the recent H5N1 outbreak that rapidly spread across flyways.

The Atlantic and Pacific oceans present a greater barrier to AIV gene flow, and AIVs are genetically segregated by the American and Eurasian landmasses into the “Americas” and “Eurasian” lineages, with only sporadic invasions from the Eastern hemisphere into the Western hemisphere (zu Dohna et al., 2009). Additional independently evolving lineages have been identified in South America (Pereda et al., 2008) and Antarctica (Hurt et al., 2014). Within these landmasses, AIVs regularly spread latitudinally along established avian migratory routes (Fries et al., 2015; Nelson et al., 2016). Viral jumps between eastern and western hemispheres are rare, but can lead to severe outbreaks (Alkie et al., 2022; Caliendo et al., 2022). Incursions of Eurasian highly pathogenic avian influenza (HPAI) of the H5N1 subtype into the Americas via wild birds led to large-scale poultry outbreaks in 2014-2015 (Saito et al., 2015; USDA APHIS, 2015; Lee et al., 2017) and again in 2021-2023 (Adlhoch et al., 2023; Caliendo et al., 2022; USDA APHIS, 2022). The ongoing 2021-2023 outbreak has killed more than 50 million US poultry, as well as large numbers of wild birds (USDA APHIS, 2023a; USDA APHIS, 2023b). The spread of highly pathogenic H5N1 viruses through wild birds and poultry in the Americas since 2021 demonstrates the importance of understanding the distinct roles of wild bird species in the evolution, maintenance, and spatial dissemination of AIV.

Gulls are opportunists adapted to a range of urban and natural environments cohabitated by wild waterfowl, humans, and poultry (Ineson et al., 2022). Of the sixteen influenza HA subtypes (H1-H16) found in wild birds, two (H13 and H16) are found almost exclusively in gulls. H16 preferentially pairs with the N3 neuraminidase in gulls, while H13 pairs with N2, N6, and N8 (Postnikova et al., 2021). Despite the presence of predominantly H13 and H16 subtypes of AIV in gulls, gulls are susceptible to HPAI H5 infection (Ramis et al., 2014; Gulyaeva et al., 2016) and have not been spared from HPAI H5 outbreaks and mortality events; for example, 94 gull mortality/morbidity events have been recorded among the 5,552 detections of HPAI H5 in wild birds in the United States (USDA APHIS, 2023a). Recent studies document that gulls serve an important ecological role in the long-distance spatial diffusion of HPAI (Hill et al., 2022). Surveillance of gull AIVs historically target marine coasts because of abundant gull numbers and increased chances of intercontinental bird movements, while inland gulls are less studied.

To address this long-standing surveillance gap, we conducted active AIV surveillance in two species of gulls (ring-billed gull, Larus delawarensis and Franklin’s gull, Leucophaeus pipixcan) that breed in Minnesota freshwater systems during summer months and visit multiple flyways and habitats during migration to obtain insights on AIV spillover, maintenance, and long-range dissemination. Our study followed a gull species (ring-billed gull) that breeds, migrates and winters in both coastal and freshwater ecosystems primarily in the U.S (Cotter et al., 2012). More specifically, we studied the Eastern population of ring-billed gulls, i.e., those breeding in locations east of the 96th meridian) (Giroux et al., 2016). We also studied a second species (Franklin’s gull) with a very different distribution. It breeds in and around lakes and marshes of the prairies of north-central North America and migrates through the center of the continent to and from wintering grounds in South America where it is primarily found in marine environments (Burger and Gochfeld, 2020). Although their distributions differ, both species have the potential to cover large expanses of the landscape in the Eastern United States. Furthermore, while both species are considered American gulls in that they breed exclusively in North America, they are distinct in several ways (Pollet et al., 2020). Ring-billed gulls are one of the most abundant and wide-ranging gull species in the interior of this region annually and therefore overlap a significant portion of poultry production in the United States (USDA ERS, 2023). In contrast, Franklin’s gulls have a more restricted distribution, are much less abundant, and considered to be one population (Burger and Gochfeld, 2020). Samples for AIV surveillance were collected at landfills during migration for both species. However, only ring-bill gulls were sampled at their nesting colonies which were populated by 5,000 to 13,000 ring-bill gull pairs (Rasmussen, 2021a). We targeted both location types for sampling because they represent idea sites to facilitate pathogen transmission among conspecifics and between species. Additionally, large numbers of birds can be easily captured at landfills and nesting colonies. Lastly, for a more complete understanding of AIV ecology, we also attached geolocators to four ring-billed gulls to track their seasonal migration movements.

## Materials and methods

### Animal care and use

All capture and sampling methods were conducted under the approval of the Institutional Animal Care and Use Committee at the University of Minnesota, Protocol # 1910-37530A. Permits to capture and band were approved by the Minnesota Department of Natural Resources and USGS, Permit # 21412-AN.

### Sample collection

Between October 2016 and August 2018 our team collected oropharyngeal and cloacal swabs from 1,566 ring-billed gulls and 120 Franklin’s gulls in Minnesota, using the methods for gull capture/release and at the locations as described in Froberg et al., 2019. Due to well-documented gull prevalence at landfills during periods of migration (Belant et al., 1995; Belant et al., 1998), six landfills were selected for the migration periods in spring (months of April and May) and fall (months of August, September, and October). Landfills were located within the Minnesota counties of Kandiyohi, Blue Earth, Cottonwood, Dakota, Kanabec, and Rice. These six sites for Franklin’s and ring-billed gulls capture and sample collection were also selected based on geospatial information from the Minnesota Turkey Growers Association and the Minnesota Board of Animal Health regarding their proximity to poultry facilities. The three, freshwater, breeding habitat, sampling locations for the breeding period in the summer months of May, June and July were spatially segregated across Minnesota—Hermit Island and Big Island on Marsh Lake in Big Stone County, Little Pelican Island on Leech Lake in Cass County, and Interstate Island Wildlife Management Area in St. Louis County. Franklin’s gulls were not sampled at their nesting locations.

To detect AIV in the swabs, the University of Minnesota Mid-Central Research and Outreach Center (Willmar, Minnesota) performed real-time reverse-transcription polymerase chain reaction (rRT-PCR) according to the following standard virus detection protocols. Briefly, viral RNA was extracted using a MagMAXTM-96 Viral RNA Isolation Kit (Applied Biosystems, Foster City, CA) following manufacturer’s instructions and using automatized robotic extraction equipment, the MagMAXTM Express-96 Deep Well Magnetic Particle Processor (Applied Biosystems). After extracting the RNA, rRT-PCR was performed following the procedures, primers, and probe described by Spackman et al. to detect the influenza A virus matrix gene (Spackman et al., 2002). A specimen was considered positive for the AIV RNA if the cycle threshold (Ct) value was less than or equal to 40. A total of 79 Franklin’s gulls and 75 ring-billed gulls were IAV rRT-PCR positive (Rasmussen, 2021b).

### Genetic sequencing

To elucidate how wild gull viruses relate to other AIVs circulating in the US and globally, we completed whole genome sequencing (WGS) on AIV rRT-PCR positive samples with Ct values < 35 from 49 ring-billed gulls and four Franklin’s gulls. Using these data we created phylogenetic trees including the ring-billed gulls and Franklin’s gulls viruses, the publicly available 2015 reassortant EA HPAI H5N2 viruses, and other AIV data from the Influenza Research Database (IRD) (Zhang et al., 2017). We selected 53 positive samples with Ct value less than or equal to 38 (based on the matrix RT-PCR described above) and submitted them to either the J. Craig Venter Institute (JCVI), La Jolla, CA (for ring-billed gull samples) or the University of Minnesota Veterinary Diagnostic Laboratory (MVDL), Saint Paul, MN (for Franklin’s gulls samples) for WGS. Similar WGS techniques were used at both JCVI and MVDL and included, but were not limited to, the following steps. First, a one-step reverse transcription-PCR amplification was performed on extracted RNA from selected samples by using SuperScript III One-Step RT-PCR system with High Fidelity Platinum Taq DNA Polymerase (Invitrogen, Life Technologies, United States) with degenerate primers (10uM MBTuni-12M and MBTuni-13) (Hoffmann et al., 2001; Zhou et al., 2009). Next, the PCR product was visually verified by gel electrophoresis for amplified rRT-PCR products (i.e., bands) of expected lengths for all eight gene segments, the quality and quantity of the RT-PCR product was checked by NanoDrop 1000 (Thermo Fisher Scientific). The PCR product was then purified by Qiagen QIAquick PCR Purification Kit (QIAGEN, United States). The sequencing library was prepared by using the Nextera DNA XT Sample Preparation Kit (Illumina, San Diego, CA, United States) and quantified by using the Quant-iT™ PicoGreen™ dsDNA Assay Kit (Invitrogen). The barcoded libraries were pooled in equimolar concentrations and sequenced in multiplex for 150 bp paired-end on Illumina NextSeq Mid-Output Mode (130 M). Alternatively, libraries (JCVI) were generated using sequence-independent single-primer amplification and sequenced using an Illumina MiSeq Instrument (2 x 300 bp) (Djikeng et al., 2008). The quality of the raw contigs released was assessed by Fast-QC (Andrews, 2010) to remove the adapter, barcode and low-quality sequences. The trimmed contigs were *de novo* assembled by Shovill (Seemann, 2019) and the consensus sequences were annotated using FLAN, the FLu ANnotator (FLAN) annotation tool (Bao et al., 2007) maintained by National Center for Biotechnology Information in GenBank (Benson et al., 2013).

### Phylogenetic analysis

Following the removal of low-quality sequences and similar sequences from the same bird, the final dataset included 40 AIV genomes from ring-billed gulls (n = 37) and franklin’s gulls (n = 3), collected during July, August, and September 2017. To place the Minnesota gull sequences in a global context, background AIV datasets were downloaded from NCBI’s Influenza Virus Resource (IVR) (Bao et al., 2008). All sequences available from gulls and from South America were included in the final dataset. Viruses from other avian hosts (e.g., waterfowl, shorebirds, and poultry) from oversampled locations in Eurasia and North America were downsampled by country, year, and subtype to achieve representative datasets. Poultry viruses were excluded from the final analysis except for small clades retained to represent major outbreaks of H5N1, H7N9, and H9N2. Gull viruses available from GISAID but not IVR were also downloaded and included in the analysis. The final background dataset included 438 viruses sampled 1950-2022 from gulls globally (including the larger data sets available from the United States and Netherlands); 206 viruses sampled from poultry (including Minnesota outbreaks of H6N1 in 2020-2021 and H5N1 in 2022); and 560 viruses sampled from other wild birds from Europe, Asia, Oceania, Africa, Antarctica, and the Americas. Sequence alignments were generated separately for each of the eight genome segments (and separately for N2, N6, and N8) and aligned using MUSCLE v3.8.3 (Edgar, 2004). Phylogenetic relationships were inferred using the maximum likelihood (ML) method available in IQTree (Nguyen et al., 2015), incorporating a general time-reversible (GTR) model of nucleotide substitution with a gamma-distributed rate variation among sites. Due to the size of the dataset, we used the high-performance computational capabilities of the Biowulf Linux cluster at the National Institutes of Health (http://biowulf.nih.gov). To assess the robustness of each node, a bootstrap resampling process was performed (1000 replicates).

### Bayesian analysis

To investigate the evolutionary origins of the distinct Gull lineage that was observed in the NP and NS ML trees (Supplementary Figures S5, S8) in finer detail, additional datasets were generated for the NP segment and NS segment (A allele only; no Gull lineage was evident in the B allele). TempEst v1.5.3 (Rambaut et al., 2016) was used to identify potential sequencing errors that substantially deviated from the linear regression of root-to-tip genetic distance against time. A time-scaled Bayesian analysis used the Markov chain Monte Carlo (MCMC) method available in the BEAST v1.10.4 package (Suchard et al., 2018), again using the Biowulf Linux cluster. A host-specific local clock (HSLC) was used to accommodate rate variation among AIV lineages (Suchard et al., 2018). Sequences were assigned to four taxon sets: Gull lineage, Americas lineage, or South America lineage; all other viruses were considered Eurasian. A GMRF Skygrid model of population growth was used, with a cutoff of 200 years (Gill et al., 2013; Hall et al., 2016). A GTR model of nucleotide substitution was used with gamma-distributed rate variation among sites. The MCMC chain was run separately five times for each of the datasets for at least 100 million iterations with subsampling every 10,000 iterations, using the BEAGLE 3 library, to improve computational performance (Ayres et al., 2019). All parameters reached convergence, as assessed visually using Tracer v.1.7.2^1^, with statistical uncertainty reflected in values of the 95% highest posterior density. At least 10 percent of the chain was removed as burn-in, and runs were combined using LogCombiner v1.10.4^2^, and a maximum clade credibility (MCC) tree was summarized using TreeAnnotator v1.10.4^3^ and visualized in FigTree v1.4.4^4^. Accession numbers for all data used in this analysis, a GISAID acknowledgement table, tree files, and XML files are available on github (https://github.com/mostmarmot/MinnesotaGullAIV).

**FIGURE 1:**
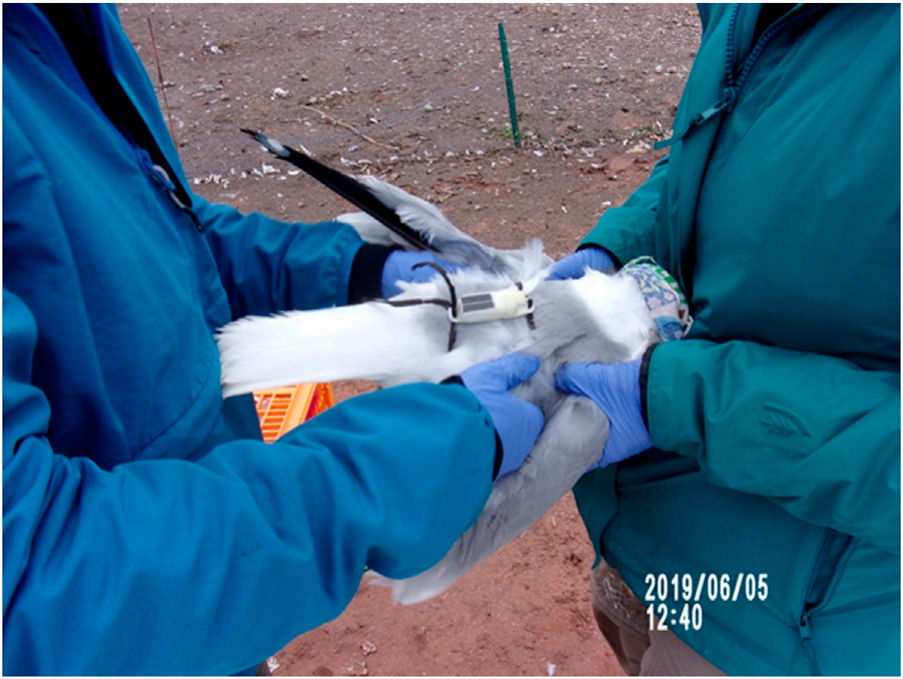
Leg-loop attachment style of GPS unit on a ring-billed gull. Image credit: Fred Strand.

GPS tracking. In addition to phylogenetic data from ring-billed gulls, we tracked ten ring-billed gulls with GPS transmitters from June 2019-April 2021 and collected resighting data from birds banded between 2016 and 2019. Briefly, in June 2019, the field team traveled to Interstate Island Wildlife Management Area in Duluth, Minnesota, a 1.9-ha ring-billed gull breeding colony in the St. Louis River. This site is occupied annually by an estimated 13,000 pairs of ring-billed gulls (Rasmussen, 2021a). Ten ring-billed gulls were captured using hand nets. Nesting disturbance was reduced by targeting different areas of the island after each bird was captured. Each gull weighed 550g or more to ensure that the GPS unit did not exceed 3% of body weight per the Guidelines to the Use of Wild Birds in Research (Fair et al., 2010). In addition to weight, body measurements were taken (head-to-beak and keel length) and each gull was banded with an alphanumeric color band and a USGS metal band. After these procedures, an Evolution Series 400 3G GSM transmitter with GPS and Accelerometer unit from Cellular Tracking Technology (CTT), Rio Grande, NJ, was affixed to each bird, using a leg-loop harness attachment style (Figure 1). The leg-loop harnesses were constructed using a 0.187″ Teflon ribbon. After the gulls were fitted with the harnesses, the units were secured with a square knot and a drop of marine-grade glue. The data plan included two GPS fixes per day with connections, i.e., data uploads to the CTT website, on Mondays and Thursdays, providing two fixes per day on a biweekly basis. Depending on a bird’s location, status, and the device’s solar charge, we obtained zero to fourteen fixes per individual, per week. Four of the ten birds provided sufficient data to be analyzed as part of this study; they were labeled 72L, 79L, 80L, and 81L. Data were collected from 1 June 2019 to 28 March 2022, for a total of 460, 557, 544, and 544 data points for birds 72L, 79L, 80L, and 81L, respectively.

### Gull banding and resighting

From October 2016 through June 2019, 1,520 ring-billed gulls were banded on the tarsus with a size 4A United States Geological Survey (USGS) aluminum leg band and a white-on-red alpha-numeric plastic leg band (Pro-Touch Engraving Ltd., Saskatoon, SK, Canada). No bands were placed on Franklin’s gulls; therefore, no information is available on their movements after they were sampled at landfills. The large alpha-numeric color bands are easily visualized by birders and even the untrained eye if a person is in close proximity to a banded bird. Resighting data were obtained through various means including team resighting efforts, personal communications to members of our team as well as reports to the USGS Bird Banding Laboratory (BBL) by citizen scientists. The BBL tracks resightings of banded birds. It is known among most birders that a bird band can be reported to the USGS BBL website. For those unfamiliar with the reporting system, an internet search of “banded bird” produces the BBL website as the first result. After submitting a banded bird report, a Certificate of Appreciation is awarded, and additional information on the bird can be requested. The total number of birds banded and encountered are publicly available for resightings after 1960. In addition, more precise information can be requested via the BBL website.

The field team also attempted to resight birds at landfills, colonies, and other ring-billed gulls loafing locations using a spotting scope in combination with binoculars to read leg bands on individual ring-billed gulls. Resighting data were summarized and compared with phylogenetic and GPS data.

## Results

**FIGURE 2:**
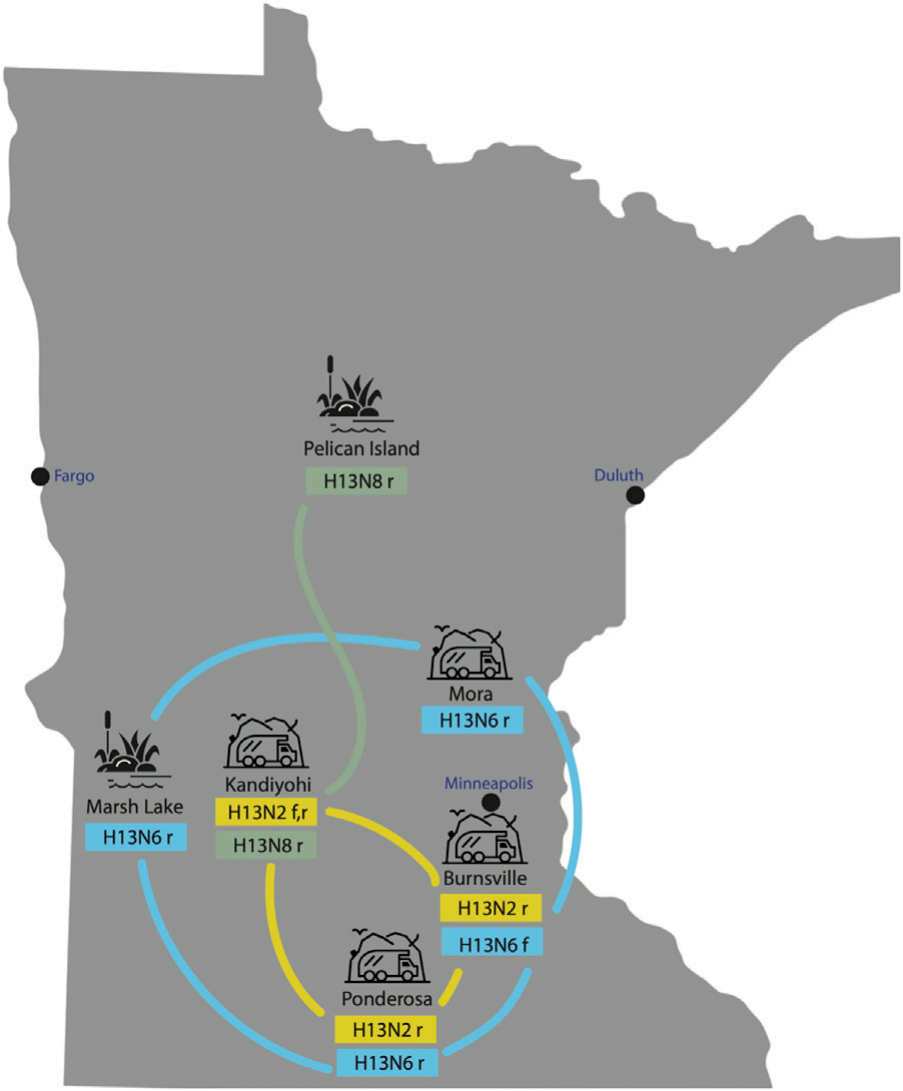
AIVs collected at six sampling sites in Minnesota. AIVs were genetically sequenced from six sampling sites in Minnesota, including two natural lake habitats and four landfills. The subtype(s) identified at each site are listed, along with the species from which the viruses were collected (r = ring-billed gull; f = Franklin’s gull). Lines indicate genetic relatedness between populations, inferred on the phylogenetic tree.

H13N2, H13N6, and H13N8 viruses identified in Minnesota gulls. All 40 AIVs sequenced from Minnesota gulls had the H13 subtype in the HA segment. Three NA subtypes were identified: H13N2 (n = 6), H13N6 (n = 10), H13N8 (n = 23). One virus was a coinfection with N2 and N6 subtypes. H13N6 and H13N8 viruses were identified in ring-billed gulls visiting natural lakes and marshes during the July summer breeding season. H13N2 and H13N6 viruses were identified in ring-billed gulls and Franklin’s gulls visiting landfills during fall migration (August-September) (Figure 2). One stray H13N8 virus was identified in the Kandiyohi landfill during late August migration (A/ring-billed-gull/Minnesota/CLMNAI1320/2017(H13N8), accession MH763874).

**FIGURE 3:**
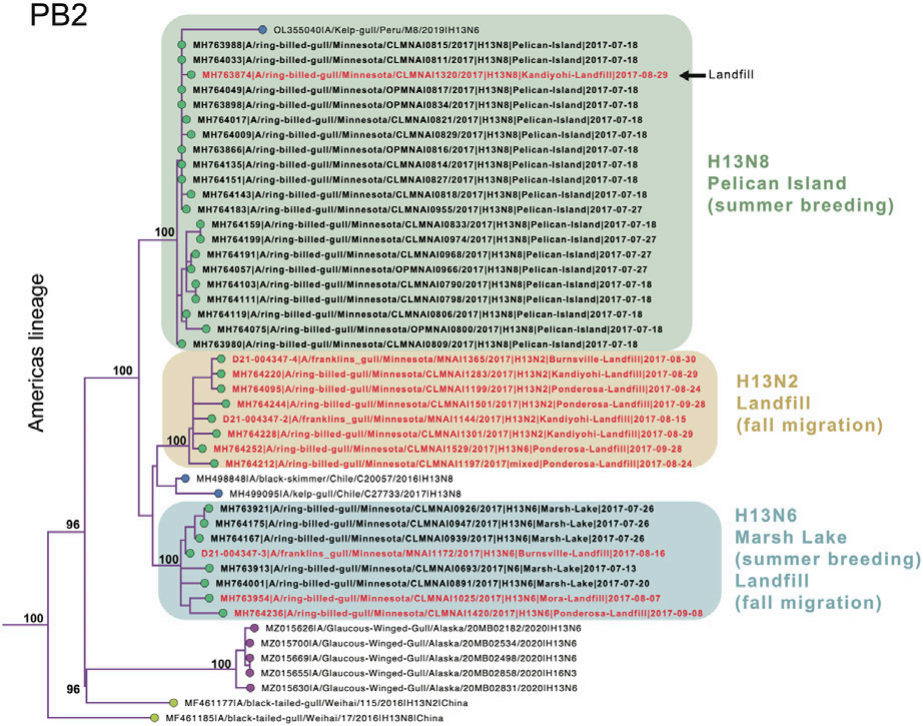
Clades of H13N2, H13N6, and H13N8 viruses. A subsection of the PB2 phylogenetic tree that contains the Minnesota gull viruses and closely related viruses from the United States, Peru, Chile, and China is presented. Circles at the tips of the tree are shaded by location (green = Minnesota; purple = other North American locations; yellow = Asia; blue = South America). Bootstrap values are presented for key nodes. The three clades of Minnesota viruses are shaded by subtype and virus names are shaded by habitat (black = natural; red = landfill).

Extensive viral gene flow among sampling sites in Minnesota gulls. Phylogenetically, Minnesota gull viruses clustered by subtype, not location or species, producing three separate clades for H13N2, H13N6, and H13N8 viruses (Figure 3; see Supplementary Figures S1–S8 for phylogenetic trees inferred for all segments). Viruses with the same subtype traveled extensively among locations and, in some cases, between summer and fall seasons. The H13N2 clade contained viruses from three landfills (Burnsville, Kandiyohi, and Ponderosa). The H13N6 clade contained viruses from Marsh Lake and three landfills (Burnsville, Mora, and Ponderosa). The H13N8 clade contained viruses predominantly from Pelican Island, as well as the stray Kandiyohi landfill virus mentioned above. The viruses observed in Minnesota gulls were genetically distinct in all segments from the H6N1 viruses that caused an outbreak in Minnesota turkeys in 2020 (Supplementary Figures S1–S8).

**FIGURE 4:**
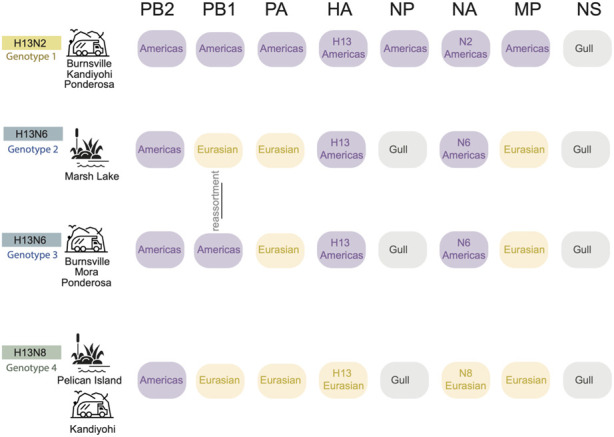
Four H13 genotypes identified in Minnesota gulls. Each row represents a genotype identified in Minnesota gulls in this study. The AIV lineage is listed for each of the eight segments of the AIV genome. Genotypes 2 and 3 differ in only the PB1 segment, which was swapped by genomic reassortment. The sampling site(s) and habitat where each genotype was identified is listed in the far right column.

Four reassortant genotypes identified in Minnesota gulls. All viruses identified in this study had reassortant genotypes with segments derived from more than one AIV lineage (Figure 4). H13N2 viruses (genotype 1) had segments from two AIV lineages, including seven segments from the Americas and NS from the ‘Gull’ lineage that circulates in gulls globally (Figure 4). Genotypes 2, 3, and 4 had segments from three AIV lineages: Americas, Eurasian, and Gull. H13N8 viruses had a 1:5:2 reassortant genotype with one segment from the Americas lineage (PB2), five Eurasian segments (PB1, PA, HA, NA, and MP) and two Gull segments (NP and NS) (genotype 4, Figure 4). The two genotypes identified among the H13N6 viruses differed by one segment, PB1 (genotypes 2 and 3, Figure 4). The H13N6 viruses clustered together on the trees inferred for the other seven segments, indicating that the new PB1 segment was acquired through reassortment locally between the summer and autumn sampling efforts. Minnesota gull viruses shared certain features; all viruses in this study had PB2 segments from the Americas lineage and NS segments (A allele) from the Gull lineage. Genotypes 2-4 shared additional features, and the vast majority of Minnesota gull viruses had a Gull NP and Eurasian PA and MP segments.

**FIGURE 5:**
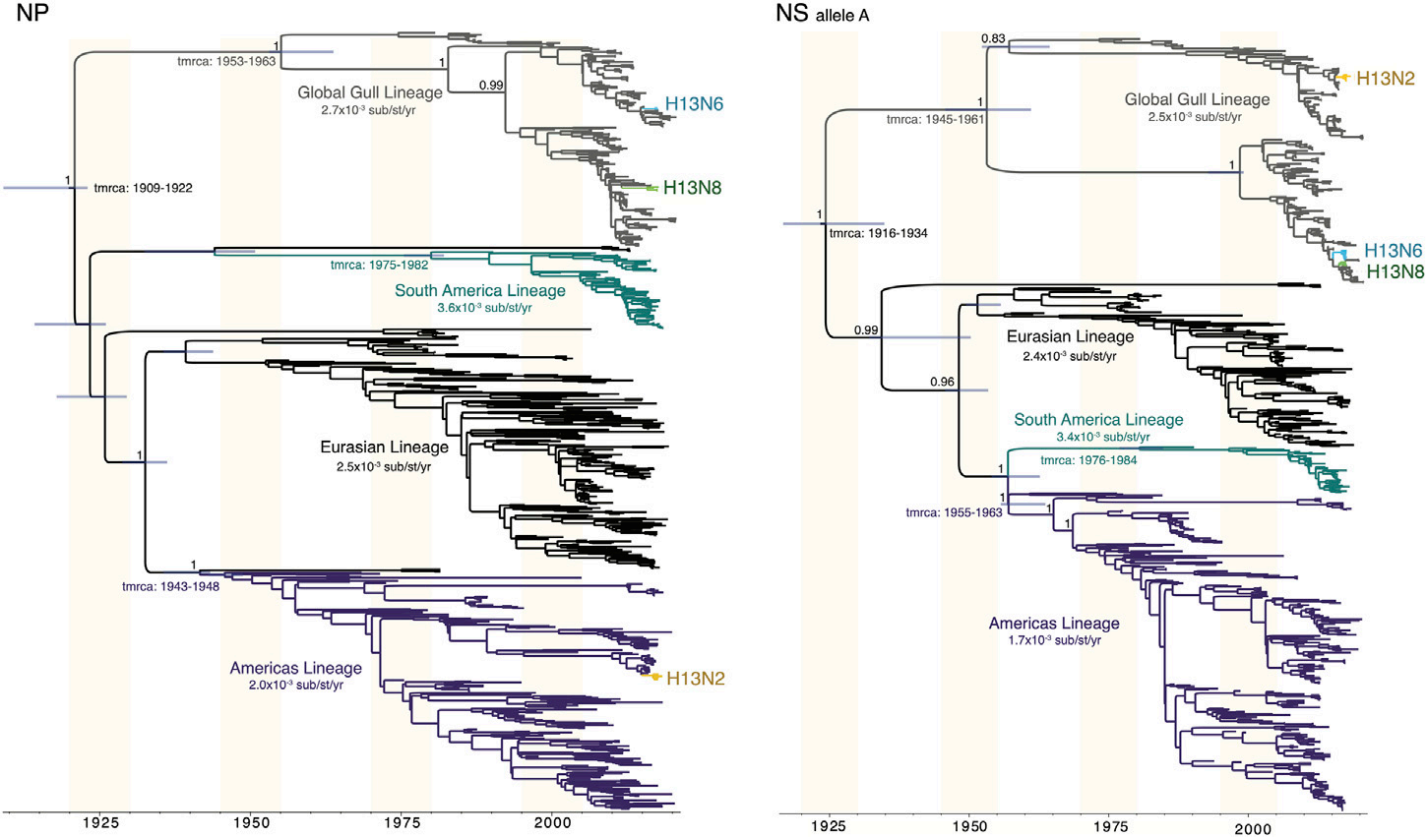
Time-scaled MCC trees of NP and NS segments of AIVs sampled globally. For both the NP segment and the NS segment (A allele), a maximum clade credibility tree depicts the evolutionary relationships and estimated times of common ancestors of four AIV lineages defined by geography (Americas, Eurasian, South America) and host species (Gull). A host-specific local clock was used to accommodate variation in the evolutionary rates among the four lineages. Clades of H13N2, H13N6, and H13N8 viruses from Minnesota are labeled.

Gull lineages in the NP and NS segments. The Gull lineage diverged from other AIV lineages during the first half of the 20th century on both the NP and NS trees (Figure 5). The Gull lineage occasionally spills over into non-gull avian species (e.g., A/ruddy turnstone/New Jersey/AI01-1407/2001(H13N6), accession MH500869), but these occurrences are rare and do not persist. Weak geographical subdivision was observed within the Gull lineage, evidence of frequent intercontinental movement. The Gull lineage splits into multiple subclades, but these were not geographically defined. Minnesota gull viruses with different subtypes did not cluster together within the same subclade (Figure 5). A host-specific local clock (HSLC) was used to accommodate rate differences among the four AIV lineages, but the Gull lineage evolved at a rate similar to the Eurasian and Americas lineages.

Comparison of AIV genotype diversity in gulls in Minnesota to other locations. The viruses observed in Minnesota gulls shared genetic similarities with viruses collected in gulls globally. The Gull lineage was similarly dominant in the NP and NS segments in the Netherlands and Alaska, United States, two locations where gulls have been intensively sampled (Verhagen et al., 2014; Reeves et al., 2018; Hill et al., 2022) (Supplementary Figure S9). The Eurasian lineage was dominant in the PA and MP in gull viruses in all three locations—Minnesota, Alaska, and the Netherlands. However, gull viruses in the Netherlands were two-lineage reassortants (Eurasian/Gull), with no evidence of the three-lineage reassortants found in Alaska and Minnesota (Supplementary Figure S10). Genotypes 2, 3, and 4, as defined in this study, were also present in Alaska gulls, but genotype 1 was not observed in North American birds outside Minnesota, only in Chilean gulls in 2016 (e.g., A/kelp gull/Chile/C8609/2016(H13N2); accession EPI_ISL_327602)).

**FIGURE 6:**
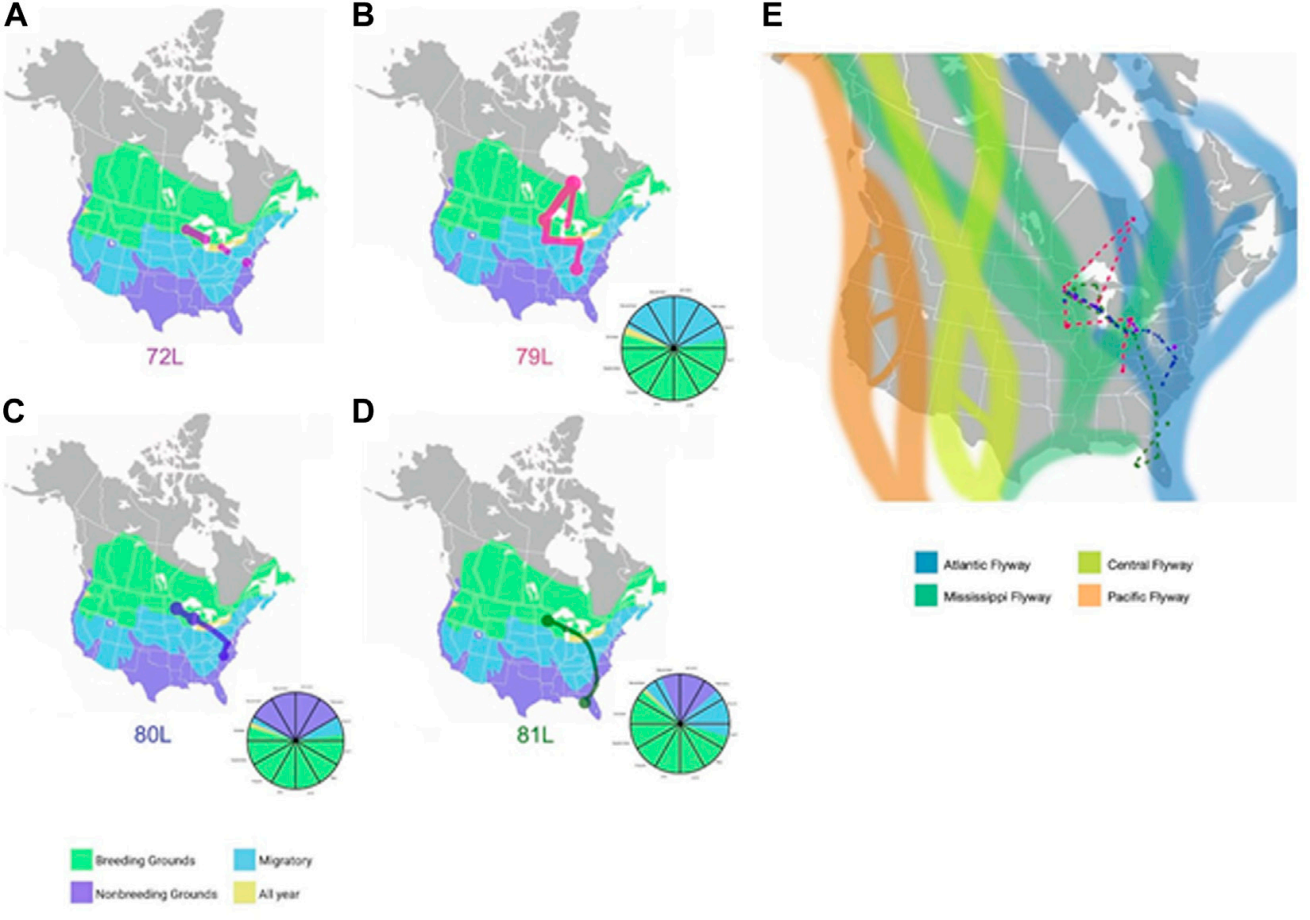
Migration routes of four ring-billed gulls that nested in Duluth, MN **(A)**. Locations detected by GPS of gull 72L for the 2019-2020 years are shown as red dots and lines against a background of the United States with areas of gull presence shaded in green (breeding grounds), blue (migratory zones), lavender (non-breeding grounds), or yellow (locations with gulls present all year). The dots represent sites the gull remained for several months and lines are the inferred migration routes. **(B)**. GPS locations detected for gull 79L during the 2019-2020 years are shown as magenta dots and lines against a background of the United States with areas of gull presence shaded in green (breeding grounds), blue (migratory zones), lavender (non-breeding grounds), or yellow (locations with gulls present all year). The dots represent sites the gull remained for several months and lines are the inferred migration routes. **(C)**. GPS locations detected for gull 80L during the 2019-2020 years are shown as blue dots and lines against a background of the United States with areas of gull presence shaded in green (breeding grounds), blue (migratory zones), lavender (non-breeding grounds), or yellow (locations with gulls present all year). The dots represent sites the gull remained for several months and lines are the inferred migration routes. **(D)**. GPS locations for gull 81L during the 2019-2020 years are shown as green dots and lines against a background of the United States with areas of gull presence shaded in green (breeding grounds), blue (migratory zones), lavender (non-breeding grounds), or yellow (locations with gulls present all year). The dots represent sites the gull stayed and lines are the inferred migration routes. The pie charts accompanying figures **(B–D)** represent the location each gull what in at the corresponding time of year. The same colors are used for location as are used in figures A, B, C, and **(D)**. Limited temporal information was detected for gull 72L, therefore a pie chart was not created. **(E)**. Dotted lines represent the migration routes for four ring-billed gulls (72L (red), 79L (magenta), 80L (blue), 81L (green)) inferred from GPS location data submitted from geolocators affixed to the animals for the 2019-2020 years. The four North American avian flyways (Pacific, Central, Mississippi, Atlantic) are shown in the background for context. Background maps in Figures 5A–D are from images obtained from Wikimedia Commons under the Public Domain Dedication. Large circles and paths were drawn to account for the uncertainty in the granular details of the gulls’ paths.

Geolocator-tracked movements of ring-billed gulls. Geolocators provided additional information about the migration routes of four ring-billed gulls that bred each summer in Duluth, Minnesota (Figure 6) during our study. Despite returning each summer to the exact breeding colony location where they were banded, none of the four birds wintered together but each consistently wintered at the same general location during our study. One ring-billed gull (72L) wintered in Maryland; 79L in Tennessee; 80L in North Carolina; and 81L in Florida. Ring-billed gull 79L flew north to extreme northern Ontario (Hudson Bay) and then moved south to spend time in the Great Lakes before overwintering in Tennessee. All four birds followed migration routes that spanned both Mississippi and Atlantic flyways.

**FIGURE 7:**
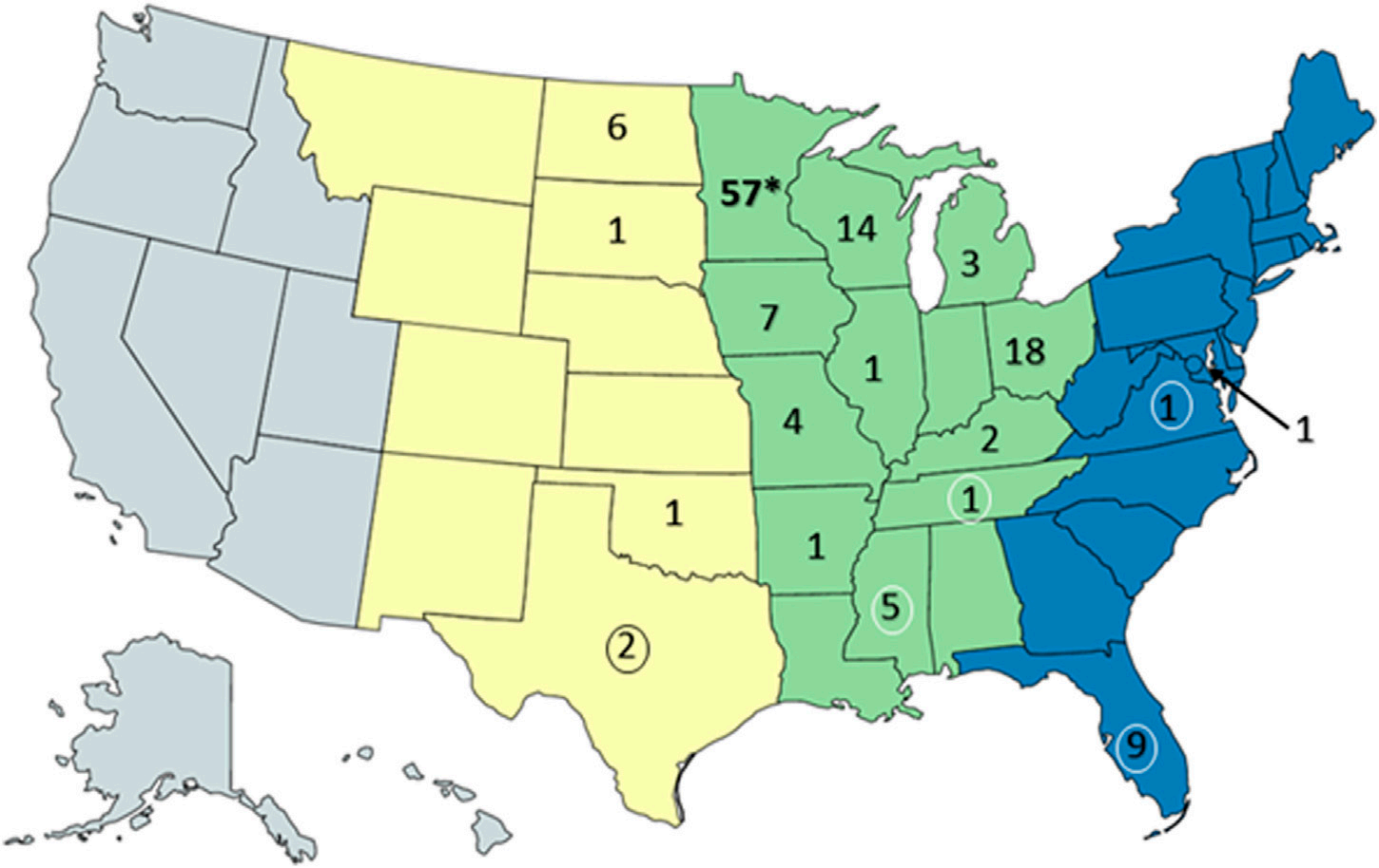
Resightings of ring-billed gulls banded in Minnesota. Observers of gull leg bands reported 134 resightings to the US Bird Banding Laboratory. This map of the United States was created with mapchart.net. The states were colored by general areas of the flyways (Central (yellow) Mississippi (green), and Atlantic (blue)) and the numbers represent the banded gulls observed and reported within the state of the resighting. Note that some birds were observed multiple times and recorded as a resighting when the observation occurred at a location and time that differed from prior resightings. The asterisk indicates the banding location of the gulls. Numbers surrounded by ovals are resightings that occurred only in the winter months of December, January, and February.

**FIGURE 8:**
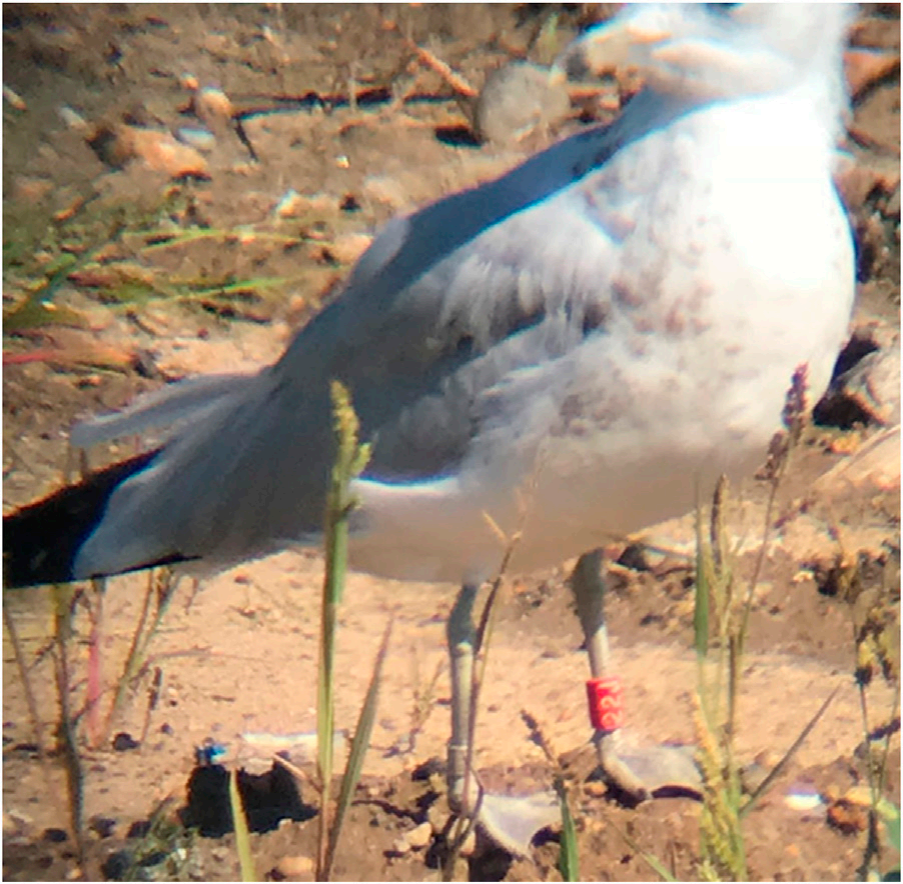
22J, Ring-billed gull resighted at a landfill in Blue Earth County, Minnesota, on 9/6/2018. This bird was originally banded at this same landfill on 9/28/17.

**FIGURE 9:**
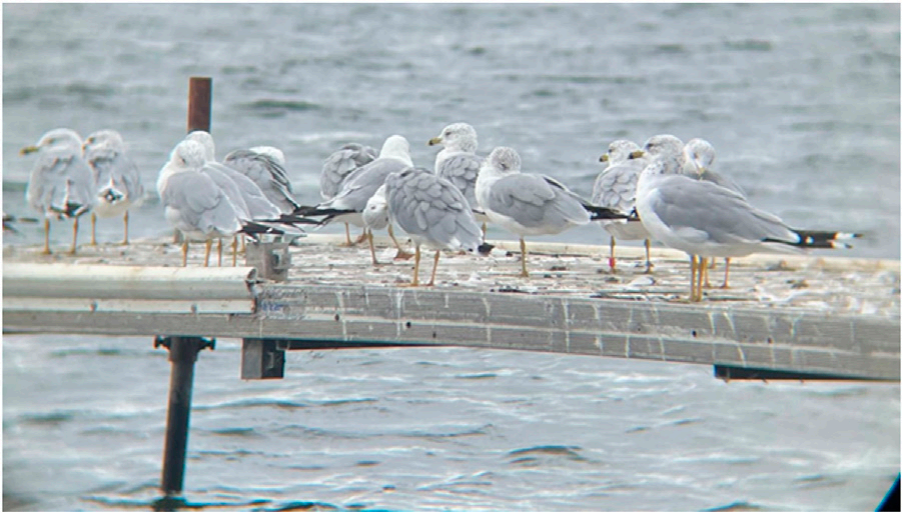
Gull 66J was re-sighted at Darling Lake, Alexandria, Minnesota on 8/27/2022. The third bird from right was captured, banded with the red, alpha-numeric leg band as shown on the left leg, and released on 9/28/2017 at a landfill 169 miles to the southeast in Blue Earth County, Minnesota.

Resightings of ring-billed gulls banded in Minnesota. As of 18 February 2023, 134 resightings of ring-billed gulls have been reported in 18 states and three flyways: 113 in the Mississippi flyway, 10 in the Central flyway, and 11 in the Atlantic flyway (Figure 7). Repeat observations of the same bird occurred, but not always in the same state or area; 88 birds were resighted only once and 18 birds were observed multiple times. It was expected that most resightings (84%) were reported in the Mississippi flyway as ring-billed gulls that nest in Minnesota are thought to follow the Mississippi river traveling south and north during fall and spring migrations, respectively (Sullivan et al., 2009). Data regarding the AIV PCR test results on cloacal (CL) and oropharyngeal (OP) swabs collected at the time of banding were available for 106 resighted birds (Table 1). For resighted birds with AIV PCR data, AIV was detected by PCR tests of the OP and/or the CL swabs of 37 (35%) of birds at the time of their banding. The AIV positive birds were not only resighted in Minnesota and in the midwestern states of Iowa, Michigan, North Dakota, and Wisconsin, but also in states that do not border Minnesota, such as Arkansas, Kentucky, Mississippi, Ohio, Tennessee and Texas. Overall, these resighting reports demonstrate that although some of the birds were found in Florida in the winter months of December, January, and February (Table 1), many more individuals were found in the Midwest (Figures 8, 9). Both AIV positive and AIV negative birds were resighted, which suggests that AIV antigen status did not seem to affect the ability of individuals to migrate from the summer breeding colony.

**TABLE 1:** Numbers of resighting reports of banding ring-billed gulls by state and month. The number of birds with AIV status determined by PCR at banding time by time and location are indicated with a number and a symbol. + = positive. - = negative. ? = unknown. * = only color of band observed. ^ = not AIV tested.

														Number of reports of individual birds by AIV status at time of banding				Total of all reports per state^a^
		Season												Total by State	Total positive	Total negative	Total unknown/not tested	
		Winter			Spring			Summer			Fall							
Flyway	State	December	January	February	March	April	May	June	July	August	September	October	November					
Atlantic	Florida	1 -	3 -											4	0	4	0	9
	Maryland				1 -									1	0	1	0	1
	Virginia	1 -												1	0	1	0	1
Central	North Dakota							2 +	1 +		1 +			4	4	0	0	6
	Oklahoma					1 -								1	0	1	0	1
	South Dakota									1 -				1	0	1	0	1
	Texas			1 +										1	1	0	0	2
Mississippi	Arkansas		1 +											1	1	0	0	1
	Iowa				1 -, 1 +						1 -	1 +	2 +	6	4	2	0	7
	Illinois									1 -				1	0	1	0	1
	Kentucky			1 ?*								1 +		2	1	0	1	2
	Michigan	1 -							1 +	1 -				3	1	2	0	3
	Minnesota						15 -	6 -	1 -	5 -, 1 +, 2 ^	5 -, 8 +	2 -	2 -	47	19	26	2	57
	Missouri		2-							-				3	0	3	0	4
	Mississippi	1 ?*	2+						1-					4	2	1	1	5
	Ohio	2-,4+		3+		1+				1 -	1 +		2 +	14	11	3	0	18
	Tennessee		1 +											1	1	0	0	1
	Wisconsin					1-					3 -, 1 -	1 -	3 -, 2 +	11	2	9	0	14
	Total	10	9	5	3	3	15	8	4	13	20	5	11	106	37	55	14	134

^a^ Some birds were resighted on multiple occasions but at different locations and on separate dates.

## Discussion

This study combines field surveillance, genetic sequencing, and geolocator tracking to understand how the varied movements of Minnesota’s inland gull populations impact the ecology and evolution of AIV. We observed high variability in migration routes among the four ring-billed gulls that were tracked with geolocators and resightings. All four birds summered in their breeding colony in Duluth, Minnesota, but each bird consistently wintered in a different state: Florida, Maryland, North Carolina, or Tennessee. One bird consistently traveled north to Hudson Bay in Ontario, Canada, which we suspect was its natal colony, before overwintering in Tennessee. Resightings data posted by citizen scientists showed that the gulls visited 18 US states spanning the Atlantic, Mississippi, and Central flyways between summer breeding seasons.

As a result, Minnesota gulls harbored reassortant viruses with genes from numerous lineages, including a) the Americas lineage dominant in US waterfowl; b) a gull-specific lineage that is sustained for the HA, NP, and NS segments; and c) the Eurasian lineage imported from the Eastern hemisphere. As a result, the potential for maintenance and evolution of the AIV in interior nesting gulls is substantial. Our study did not find any genetic linkage between Minnesota’s gull viruses and a H6N1 outbreak in Minnesota turkeys several years later, but our small sample size and the low availability of AIV sequences from poultry during the year of our study represents a significant limitation. Overall, we find that gulls in Minnesota frequently exchange AIVs with each other locally as well as acquire geographically diverse genes during migration, creating a melting pot for AIV diversity that merits further surveillance. Our findings suggest that continued surveillance of gulls during the current H5N1 outbreak will be useful in understanding how the virus evolves, reassorts, spreads long distances, and spills back and forth between poultry and wild birds. A major outstanding question is whether gulls were involved in the transatlantic spread of the current H5N1 outbreak from Europe to North America, given that one of the first H5N1 viruses identified from North America (November 2021) was from a great black-backed gull in Newfoundland, Canada (Caliendo et al., 2022). More recently, large mortality events of H5N1 in black-headed gulls in Europe in early 2023 (EFSA, 2022) raise additional questions about the role of gulls in the H5N1 outbreak as it continues to evolve and potentially reassort with gull lineages.

One basic science question that remains unresolved is why a genetically distinct gull-specific lineage in the NP and NS has evolved independently in gulls and been maintained for more than half a century without sustained transmission in non-gull species (Wille and Holmes, 2020). Experimental studies are needed to determine whether the Gull lineage confers any fitness advantage in gulls, as there does not appear to be an ecological barrier. Waterfowl share habitat with gulls and frequently exchange polymerase and matrix protein genes. Gull-specific fitness advantages in certain genes could relate to gull physiology, such as sialic acid receptor distribution (Franca et al., 2013) or immunological responses (Criado et al., 2021). Attempts to elucidate the immunopathology of gull origin H13 and H16 virus infections of non-gull hosts with experimental challenge studies are few (Brown et al., 2012; Lindskog et al., 2013). Gull-specific lineages also have a different ecology from AIV in waterfowl, reflecting how far gull viruses travel. The gull NP and NS lineages, like the H13 and H16 gull subtypes, exhibit weaker geographical structure compared to their counterparts in waterfowl, with frequent spatial mixing of gull viruses across the Western and Eastern hemispheres in both directions (east-to-west and west-to-east).

At first, we were surprised that the Eurasian lineage was frequently detected in gulls in Minnesota. The central region of the United States is located hundreds of miles from either the Atlantic or Pacific oceans. But this finding turns out to be consistent with the very high background frequency of long-distance virus gene flow observed in gulls globally in our phylogenetic trees. Whereas AIV in waterfowl is strongly spatially structured into Eastern and Western hemisphere lineages, AIV in gulls is substantially less segregated by continent, as was suggested in the 2010s when researchers revealed intercontinental AIV gene mixing in gulls in Alaska, United States (Wille et al., 2011), Newfoundland, Canada (Hall et al., 2013; Huang et al., 2014), and Iceland (Dusek, et al., 2014). We were also surprised by the post-breeding dispersal movements of gull (79L) to a gull colony site in Hudson Bay after nesting on Lake Superior (Interstate Island). We speculate that the Ontario site was its natal colony and the gull was willing to travel long distances along old familiar routes prior to wintering on Lake Erie near Cleveland, Ohio. Ring-billed gulls may return to their natal colony to breed, but usually nest at alternative sites in the same general area (e.g., lake) where they hatched (Dexheimer and Southern. 1974; Southern, 1977). Because the lifespan of ring-billed gulls can be several decades, movement over the landscape of the interior Eastern US and interactions with other gulls can be frequent and widespread as ring-billed gulls crisscross several flyways. Such movements may be seen by some as unexpected when they occur outside of prescribed migration times or when only examined in the context of man-made flyway designations. The flyway designations may get redrawn in upcoming years as larger and more integrated datasets on bird movements inform better surveillance and disease management strategies (Buhnerkempe et al., 2016). As technological advancements make sensors cheaper, smaller, and less intrusive to the animal (Dezfuli et al., 2022), tracking data has exploded. In response, the “Movebank” repository hosted by the Max Planck Institute of Animal Behavior provides an online platform for research and wildlife management communities to exchange, analyze, and archive animal tracking and other sensor data (Kays et al., 2022). In the future, as datasets get larger, it may be possible to connect how complicated migration patterns, such as those observed here in ring-billed gulls, are influenced by changes in climate or habitat availability. Going forward, there is an urgent need to harness new technologies in genomics and animal tracking to understand the role of gulls in AIV outbreaks that threaten wildlife, poultry production, and public health.

## Data Availability

The datasets presented in this study can be found in online repositories. The names of the repository/repositories and accession number(s) can be found below: GenBank OQ691603-OQ691626, MH763859-MH763909, MH763911-MH763988, MH763994- MH764260, and OQ770383-OQ771601.

## Footnotes

1
https://github.com/beast-dev/tracer/releases/latest.

2
https://beast.community/logcombiner.

3
https://beast.community/treeannotator.

4
https://github.com/rambaut/figtree/releases/tag/v1.4.4.
