# Supplementary material for: Influenza A viruses in gulls in landfills and freshwater habitats in Minnesota, United States
Source: Front Genet. 2023 May 9;14:1172048. doi: 10.3389/fgene.2023.1172048 (PMC10203411; doi:10.3389/fgene.2023.1172048)
Supplement: Supplementary file 1 [file Presentation1.pdf]

Supplementary Materials

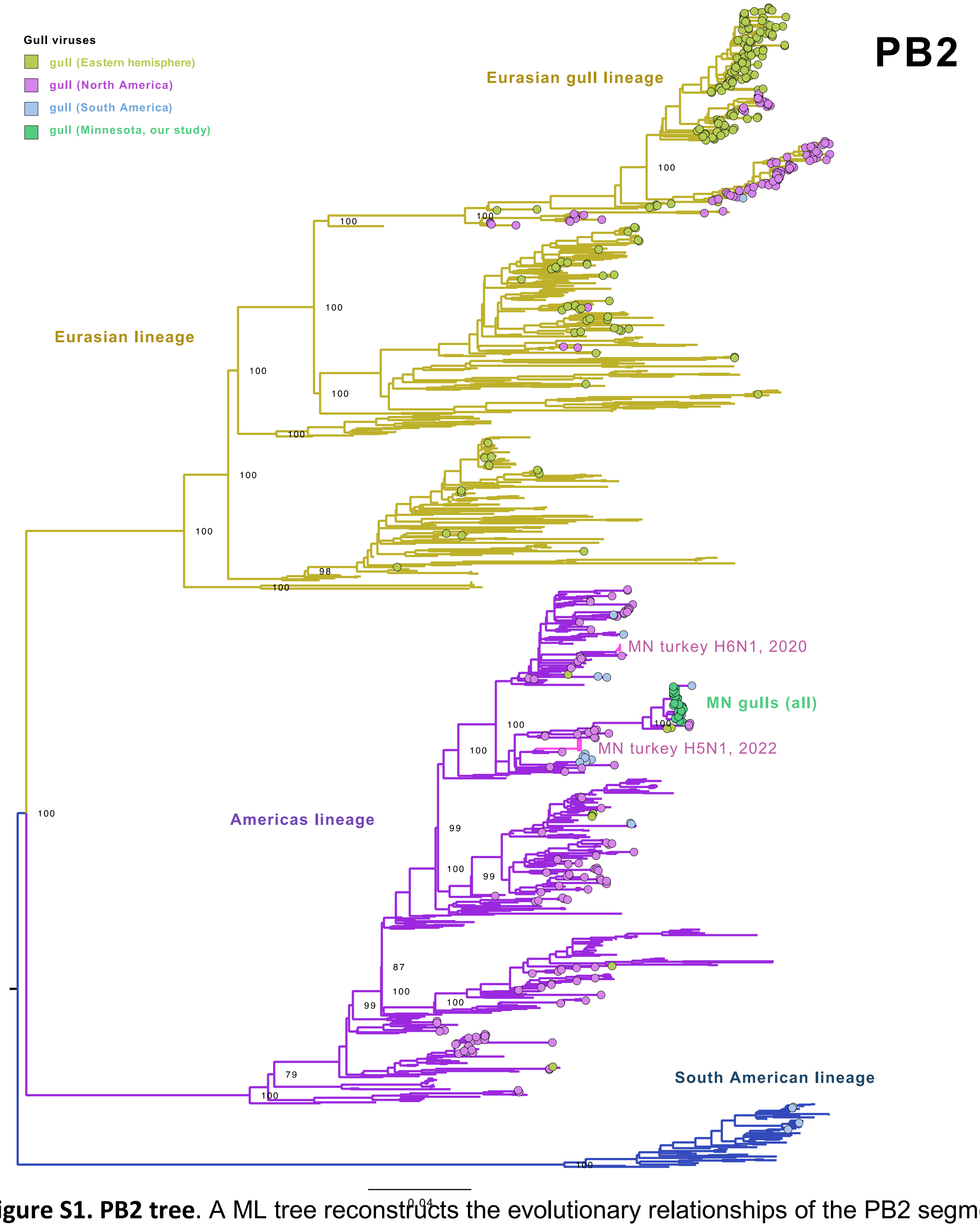

Figure S1. PB2 tree. A ML tree reconstructs the evolutionary relationships of the PB2 segment from AIVs collected globally in gulls, other wild birds, and poultry. Branches are shaded by lineage (Eurasian, Americas, South America). Each gull virus is represented by a circle that is shaded by location (Eastern hemisphere, North America, South America, or Minnesota sites included in our study). Minnesota turkey outbreaks of H5N1 and H6N1 are labeled and the branches are shaded pink. Branch lengths are drawn to scale. Bootstrap values are provided for key nodes. Raw tree files are available in the GitHub repository (https://github.com/mostmarmot/MinnesotaGullAIV)

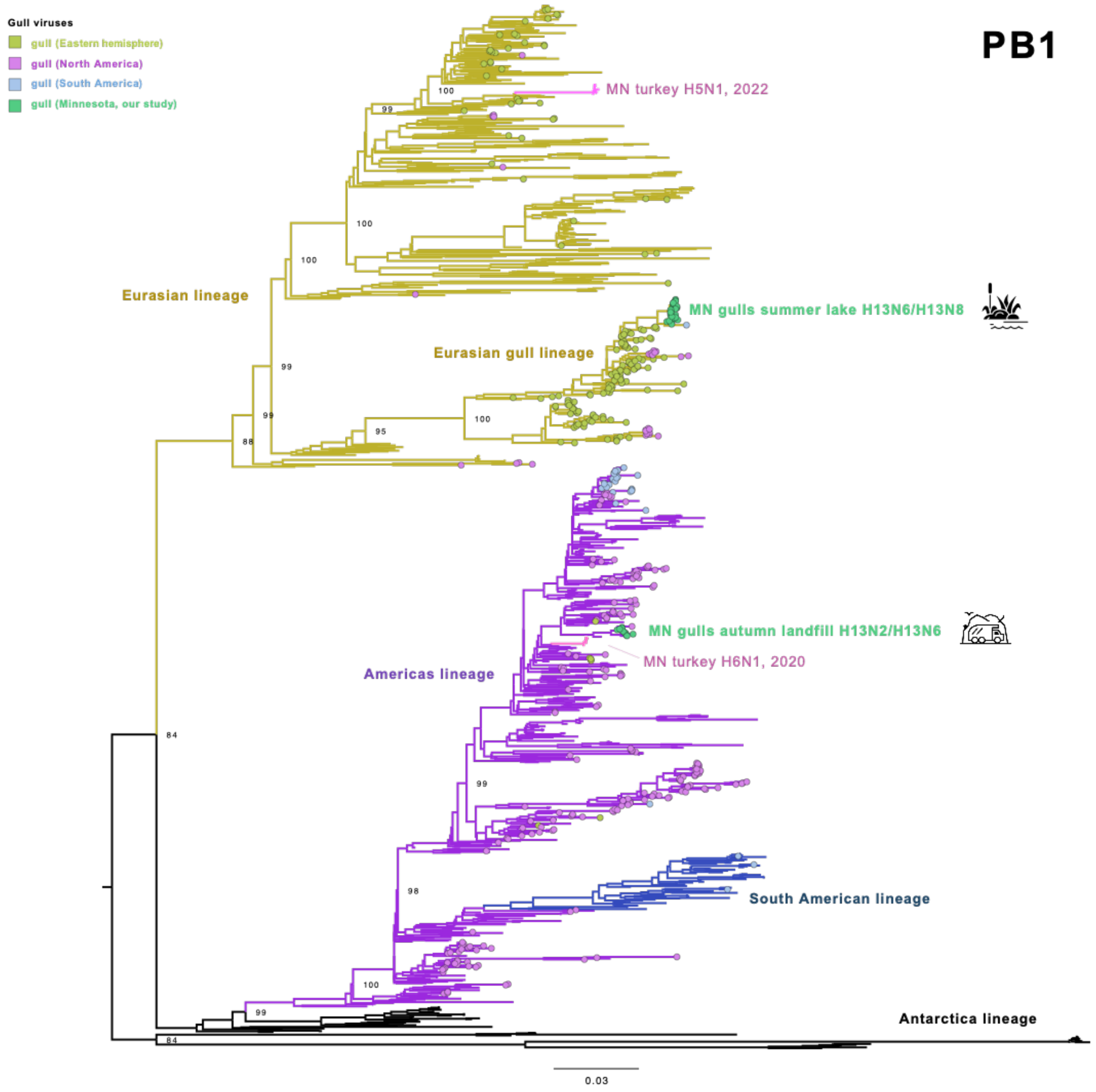

Figure S2. PB1 tree. A ML tree reconstructs the evolutionary relationships of the PB1 segment from AIVs collected globally. Branches are shaded by lineage (Eurasian, Americas, South America, Antarctica). Each gull virus is represented by a circle that is shaded by location (Eastern hemisphere, North America, South America, or Minnesota sites included in our study). Minnesota gull clades are labeled, with subtype, and cartoons indicate whether Minnesota viruses were collected in freshwater or landfill habitats. Minnesota turkey outbreaks are labeled and shaded pink. Branch lengths are drawn to scale. Bootstrap values are provided for key nodes.

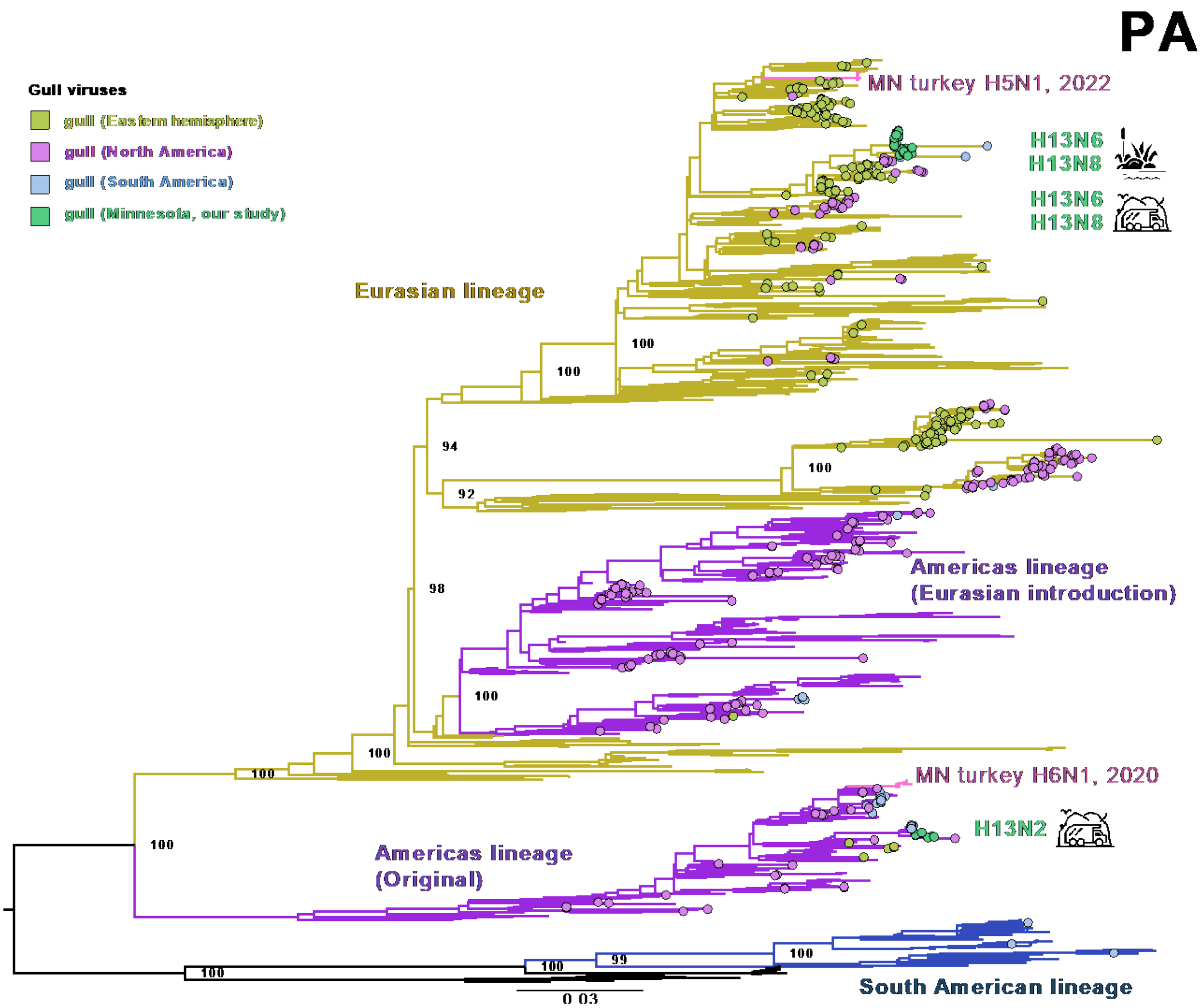

Figure S3. PA tree. A ML tree reconstructs the evolutionary relationships of the PA segment from AIVs collected globally. Branches are shaded by lineage (Eurasian, Americas, South America, Antarctica). Two Americas lineages are identified, including the original lineage and a later introduction from Europe. Each gull virus is represented by a circle that is shaded by location (Eastern hemisphere, North America, South America, or Minnesota sites included in our study). Minnesota gull clades are labeled, with subtype, and cartoons indicate whether Minnesota viruses were collected in freshwater or landfill habitats. Minnesota turkey outbreaks are labeled and shaded pink. Branch lengths are drawn to scale. Bootstrap values are provided for key nodes.

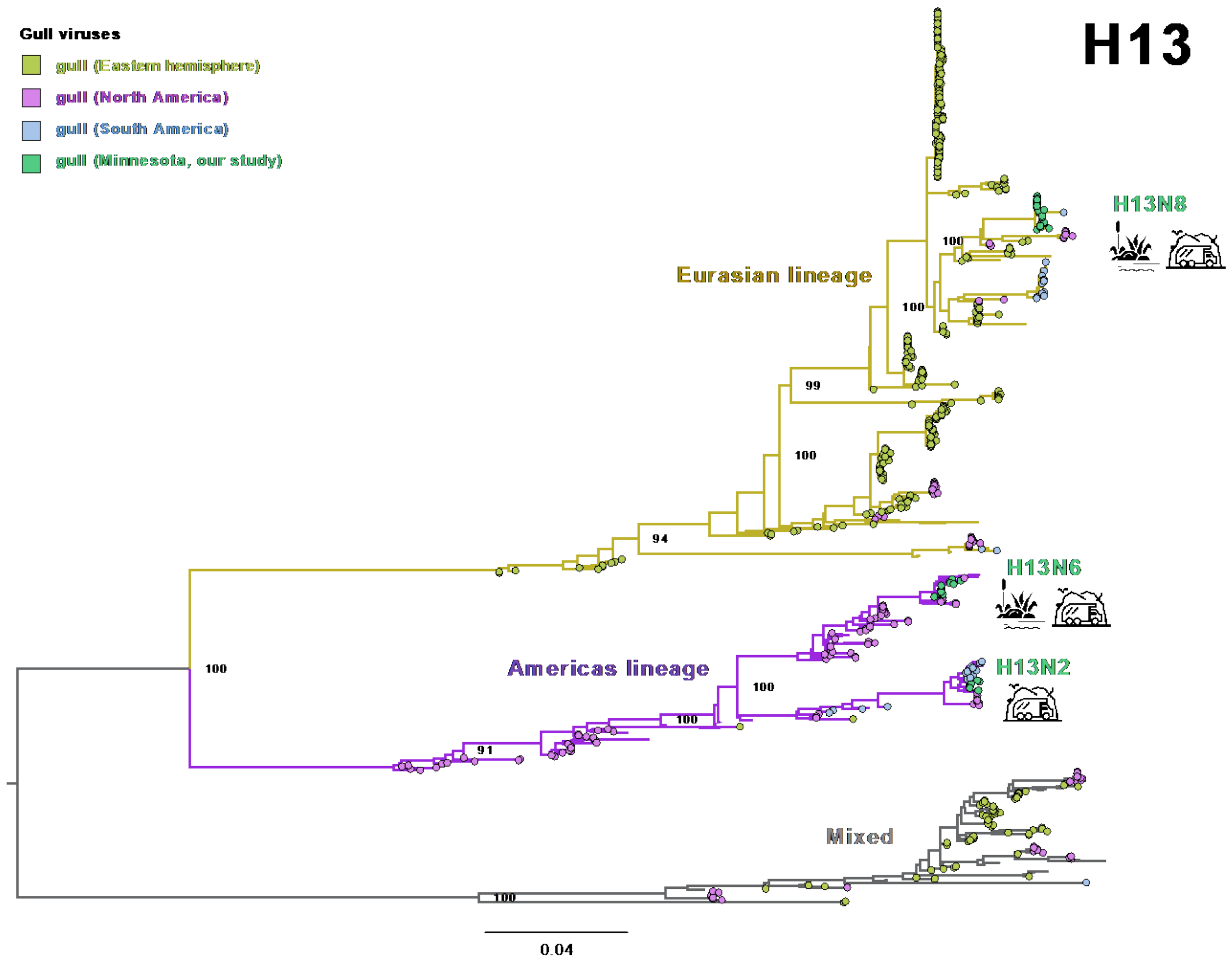

Figure S4. H13 tree. A ML tree reconstructs the evolutionary relationships of the HA (H13) segment from AIVs collected globally. Branches are shaded by lineage (Eurasian, Americas, Mixed/International). Each gull virus is represented by a circle that is shaded by location (Eastern hemisphere, North America, South America, or Minnesota sites included in our study). Minnesota gull clades are labeled, with subtype, and cartoons indicate whether Minnesota viruses were collected in freshwater or landfill habitats. Branch lengths are drawn to scale. Bootstrap values are provided for key nodes.

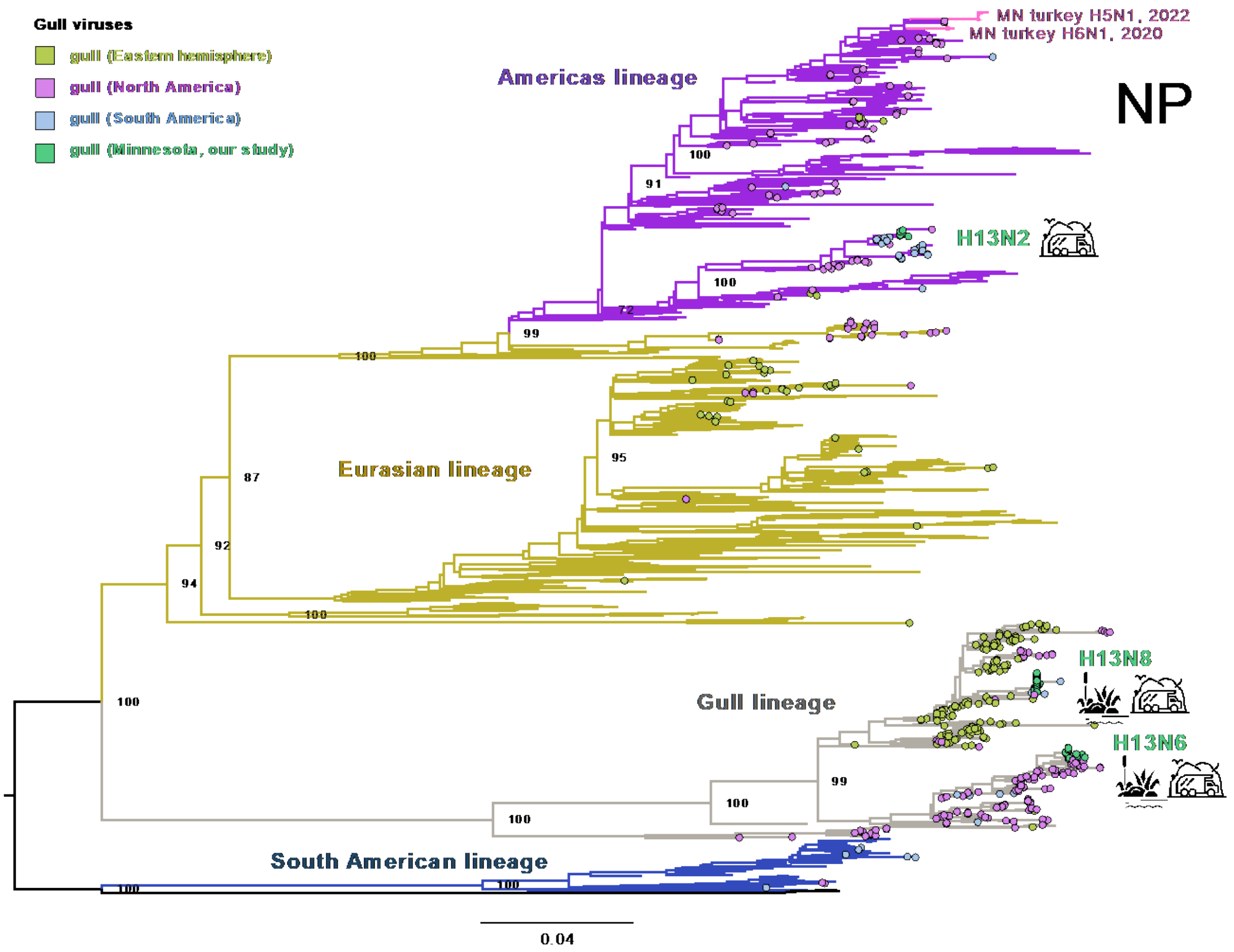

Figure S5. NP tree. A ML tree reconstructs the evolutionary relationships of the NP segment from AIVs collected globally. Branches are shaded by lineage (Eurasian, Americas, South American, Gull). Each gull virus is represented by a circle that is shaded by location (Eastern hemisphere, North America, South America, or Minnesota sites included in our study). Minnesota gull clades are labeled, with subtype, and cartoons indicate whether Minnesota viruses were collected in freshwater or landfill habitats. Branch lengths are drawn to scale. Bootstrap values are provided for key nodes.

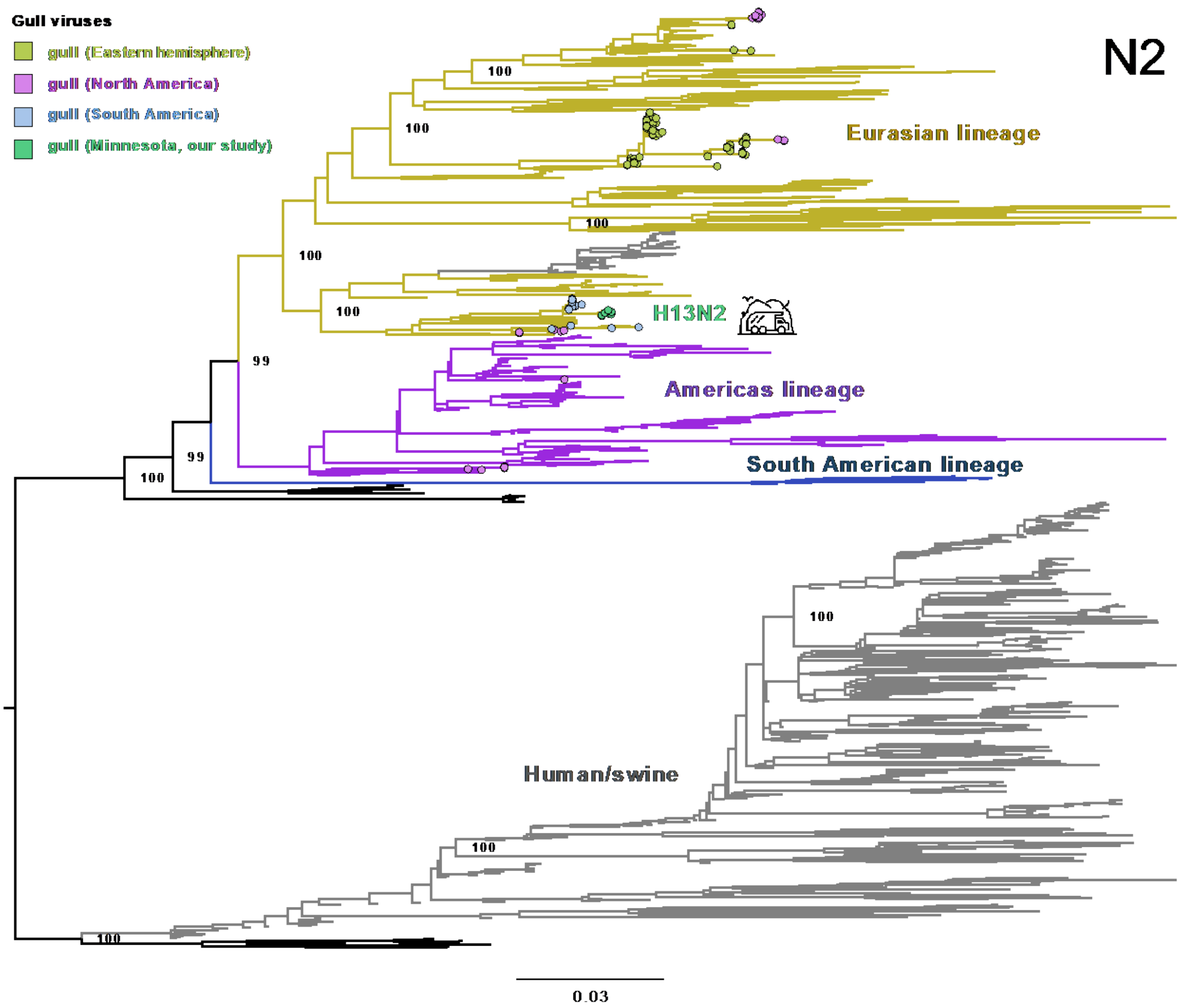

Figure S6. N2 tree. A ML tree reconstructs the evolutionary relationships of the NA (N2) segment from AIVs collected globally, with human and swine viruses included as background context. Branches are shaded by lineage (Eurasian, Americas, South American). Each gull virus is represented by a circle that is shaded by location (Eastern hemisphere, North America, South America, or Minnesota sites included in our study). Minnesota gull clades are labeled, with subtype, and cartoons indicate whether Minnesota viruses were collected in freshwater or landfill habitats. Branch lengths are drawn to scale. Bootstrap values are provided for key nodes.

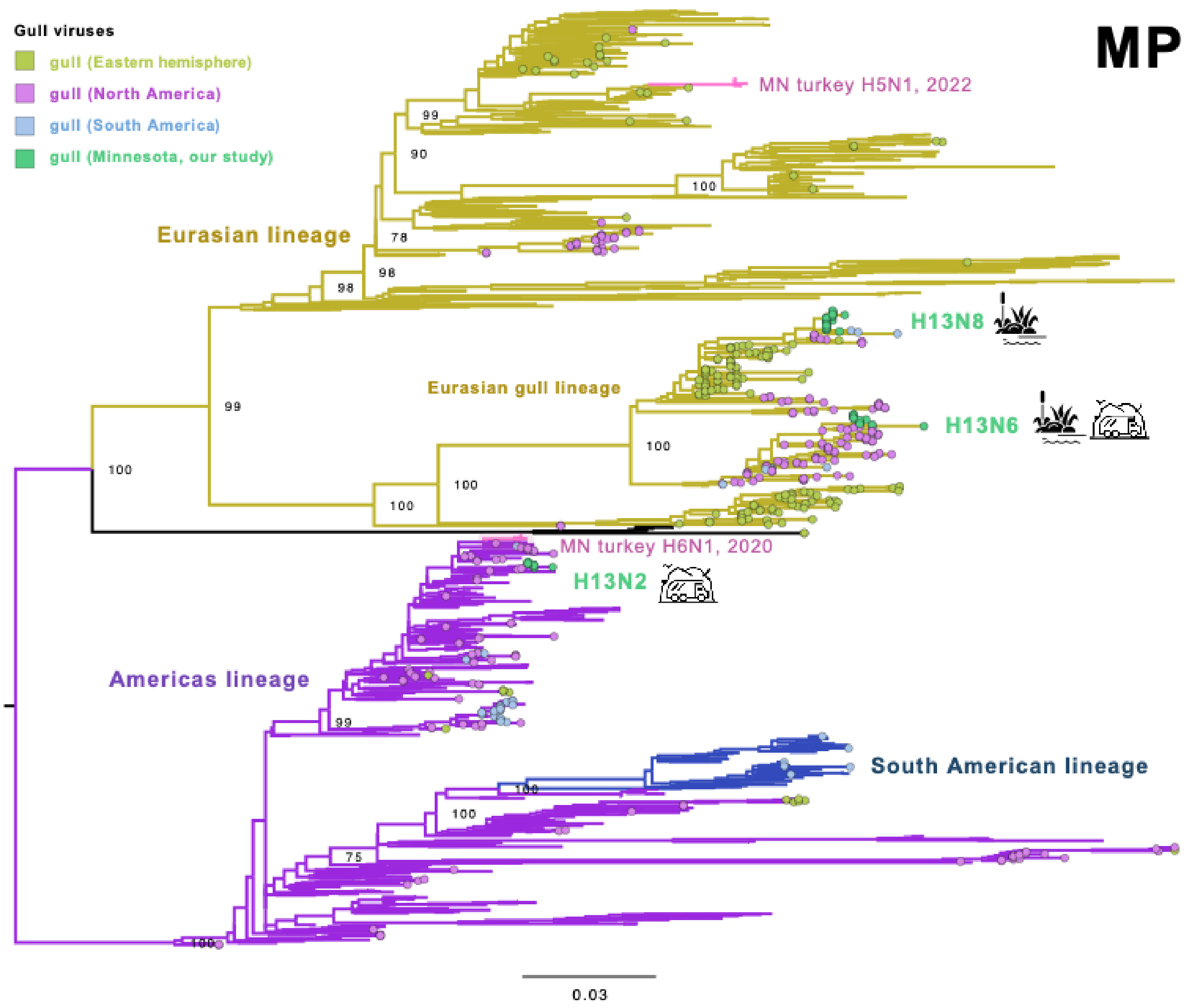

Figure S7. MP tree. A ML tree reconstructs the evolutionary relationships of the MP segment from AIVs collected globally. Branches are shaded by lineage (Eurasian, Americas, South American). Each gull virus is represented by a circle that is shaded by location (Eastern hemisphere, North America, South America, or Minnesota sites included in our study). Minnesota gull clades are labeled, with subtype, and cartoons indicate whether Minnesota viruses were collected in freshwater or landfill habitats. Branch lengths are drawn to scale. Bootstrap values are provided for key nodes.

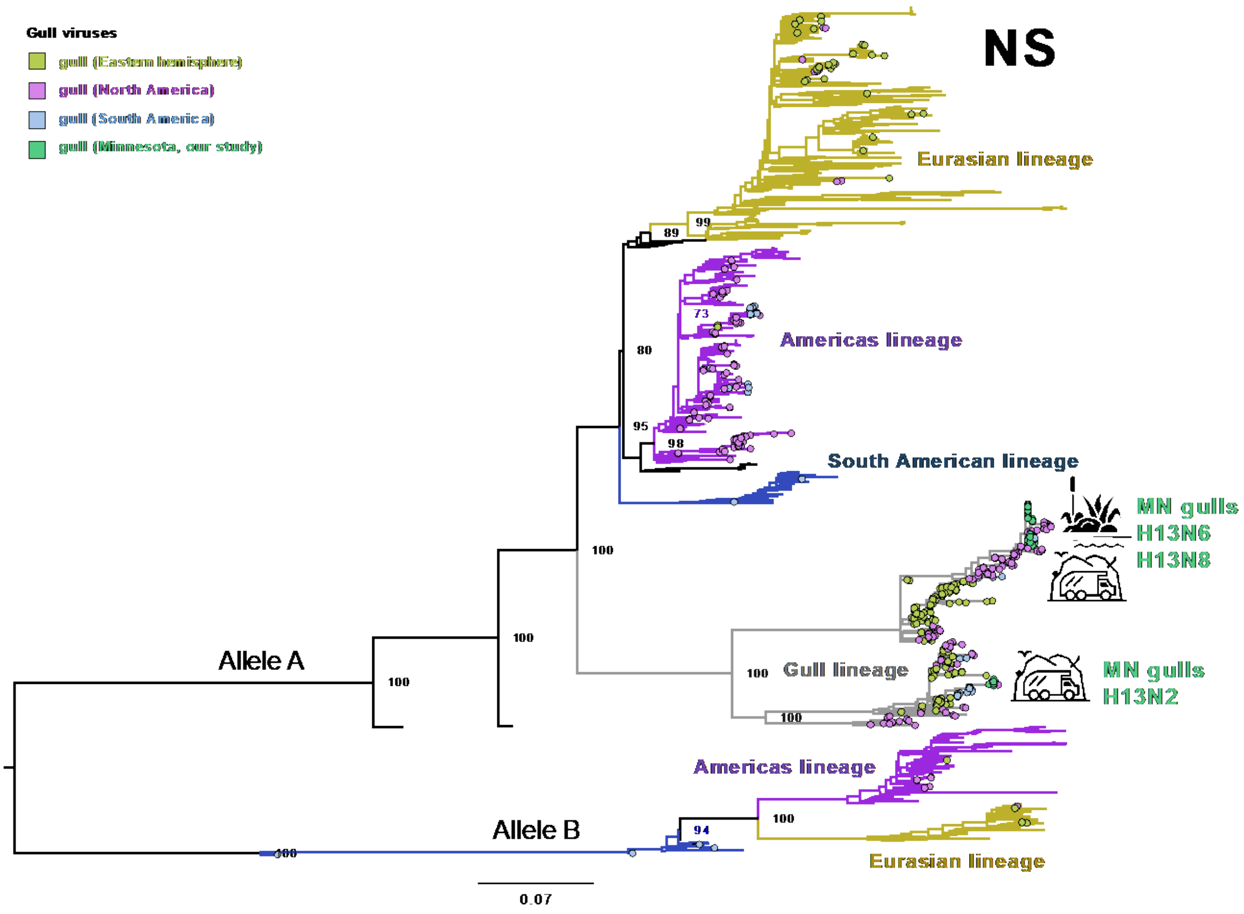

Figure S8. NS tree. A ML tree reconstructs the evolutionary relationships of the NS segment from AIVs collected globally. Alleles A and B are labeled. Branches are shaded by lineage (Eurasian, Americas, South American, Gull). Each gull virus is represented by a circle that is shaded by location (Eastern hemisphere, North America, South America, or Minnesota sites included in our study). Minnesota gull clades are labeled, with subtype, and cartoons indicate whether Minnesota viruses were collected in freshwater or landfill habitats. Branch lengths are drawn to scale. Bootstrap values are provided for key nodes.

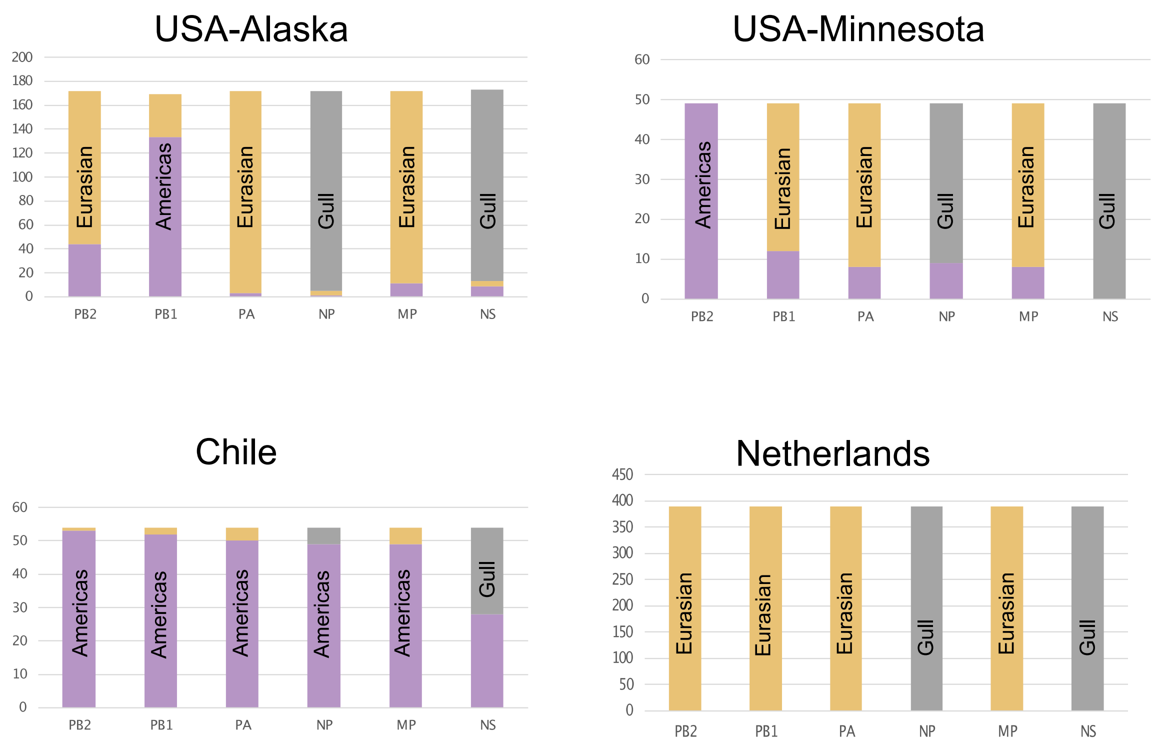

Figure S9. Lineages found in gull AIVs. The number of genetic sequences assigned to each lineage (Eurasian, Americas, Gull) is presented for each of the eight segments of the IAV genome for four locations where gulls have been relatively well sampled.

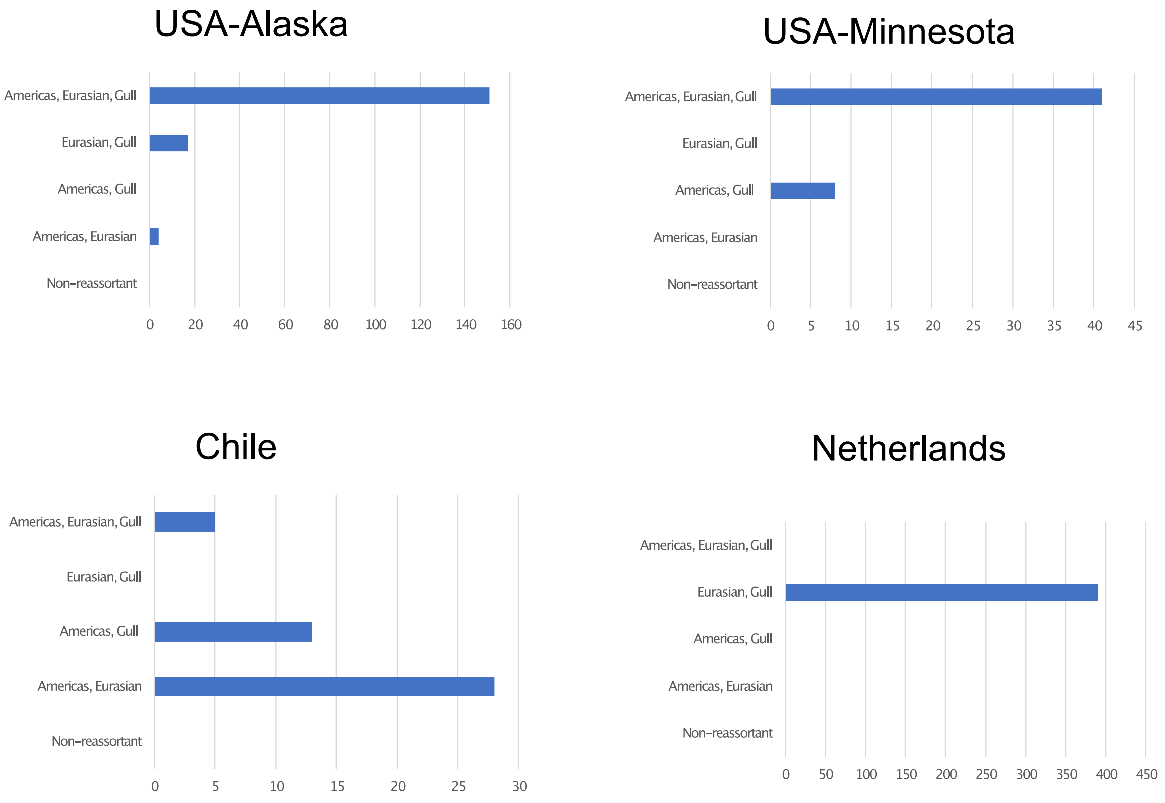

Figure S10. Reassortants found in gulls. The number of virus genomes from each reassortment category, based on the combination of lineages of individual segments, is presented for four locations where gulls have been relatively well sampled.
